# Immunotherapy Combined with Large Fractions of Radiotherapy: Stereotactic Radiosurgery for Brain Metastases—Implications for Intraoperative Radiotherapy after Resection

**DOI:** 10.3389/fonc.2017.00147

**Published:** 2017-07-24

**Authors:** Carsten Herskind, Frederik Wenz, Frank A. Giordano

**Affiliations:** ^1^Medical Faculty Mannheim, Department of Radiation Oncology, Universitätsmedizin Mannheim, Heidelberg University, Mannheim, Germany; ^2^Cellular and Molecular Radiation Oncology Laboratory, Medical Faculty Mannheim, Department of Radiation Oncology, Universitätsmedizin Mannheim, Heidelberg University, Mannheim, Germany; ^3^Translational Radiation Oncology, Department of Radiation Oncology, Universitätsmedizin Mannheim, Heidelberg University, Mannheim, Germany

**Keywords:** brain metastases, immune therapy, radiotherapy, stereotactic radiosurgery, intraoperative radiotherapy

## Abstract

Brain metastases (BM) affect approximately a third of all cancer patients with systemic disease. Treatment options include surgery, whole-brain radiotherapy, or stereotactic radiosurgery (SRS) while chemotherapy has only limited activity. In cases where patients undergo resection before irradiation, intraoperative radiotherapy (IORT) to the tumor bed may be an alternative modality, which would eliminate the repopulation of residual tumor cells between surgery and postoperative radiotherapy. Accumulating evidence has shown that high single doses of ionizing radiation can be highly efficient in eliciting a broad spectrum of local, regional, and systemic tumor-directed immune reactions. Furthermore, immune checkpoint blockade (ICB) has proven effective in treating antigenic BM and, thus, combining IORT with ICB might be a promising approach. However, it is not known if a low number of residual tumor cells in the tumor bed after resection is sufficient to act as an immunizing event opening the gate for ICB therapies in the brain. Because immunological data on tumor bed irradiation after resection are lacking, a rationale for combining IORT with ICB must be based on mechanistic insight from experimental models and clinical studies on unresected tumors. The purpose of the present review is to examine the mechanisms by which large radiation doses as applied in SRS and IORT enhance antitumor immune activity. Clinical studies on IORT for brain tumors, and on combined treatment of SRS and ICB for unresected BM, are used to assess the safety, efficacy, and immunogenicity of IORT plus ICB and to suggest an optimal treatment sequence.

## Introduction

Brain metastases (BM) are an advanced-stage manifestation of cancer that affect up to a third of patients with systemic disease. BM predominantly originate from primary lung, breast, or gastrointestinal cancers and melanoma. Given the change in demographics in industrialized countries with increasing cancer frequencies, combined with the increase in numbers of long-term survivors owing to improved diagnostics and therapy, the incidence is believed to rise further. Depending on the clinical condition, treatment options for BM include surgery, whole-brain radiotherapy (WBRT), stereotactic radiosurgery (SRS), or a combination of these, while chemotherapy has only limited activity owing to low penetration of the blood–brain barrier (BBB). A considerable proportion of patients undergo upfront surgery for debulking the tumor mass or for the determination of histology and/or mutational status. In such cases, local relapse occurs in roughly 60% of patients 1–6 months after surgery alone (1), indicating that tumor stem cells capable of forming recurrent tumors have microscopically invaded the borders of the surgical cavity. While some degree of local control may be achieved by adding WBRT, this is associated with high morbidity and intracranial recurrences are common. Randomized phase III trials did not show improved overall survival by adding adjuvant WBRT (2, 3) and most patients now undergo SRS directed to the tumor bed, a procedure that was proposed and developed even before these trials were done (4, 5). Although level I evidence for this treatment is lacking, initial data suggest a low toxicity profile (6–8). However, even with the best treatment available, the median survival is rarely much longer than 1 year and, thus, there is a strong need for improved treatment beyond the BM and the tumor bed around the excised cavity.

Similar to SRS, intraoperative radiotherapy (IORT) to the cavity and margins treats the tumor site while minimizing dose to the surrounding normal tissue. Early clinical studies on IORT after the resection of glioma were conducted especially in Japan and in Germany, typically applying 15–25 Gy of high-energy electrons in a single fraction. Results were encouraging, with comparable or better local control and overall survival, and less radionecrosis than after fractionated WBRT with external X-ray beams (9–12). A large retrospective study of IORT as a boost combined with external beam WBRT versus WBRT alone did not show any significant improvement by IORT (13). However, failures were found to be associated with insufficient dose coverage (14) and a case of long-term (9 years) survival was indeed observed (15). Because of technical limitations, few centers were able to pursue this treatment at the time, but in the last decade, dedicated mobile machines for delivering IORT by high-energy electrons or low-energy X-rays (LEX) in the operating room have become more widely available.

Compared to SRS for resected metastases, IORT eliminates the healing time between surgery and the beginning of RT during which tumor cells may proliferate and possibly spread beyond the tumor bed. In contrast, IORT requires the total dose to be applied in a single fraction, whereas hypofractionated treatment is optional with SRS (e.g., for larger tumors or cavities). Recently, the potential use of IORT for brain tumors may be supported by encouraging results from a phase I/II trial on IORT with 50 kV X-rays for glioblastoma (16), which was found to be safe in these patients (Giordano et al., submitted) and prompted the initiation of a randomized phase III trial (NCT02685605). The rationale for IORT in glioblastoma has been reviewed by Giordano et al. (17). Notably, the treatment of solitary BM with excision and IORT alone using 50 kV X-rays has been shown to be feasible with disease-specific outcome comparable to other modalities (18).

It has been suggested that in addition to targeted cell killing induced by conventional fraction sizes, vascular, cohort (bystander), and immune effects may contribute to the biological effect of very large doses per fraction (19–22). In contrast, it has been disputed whether additional effects other than the 5R’s of radiotherapy (reassortment, repair, reoxygenation, repopulation, and radiosensitivity) need to be invoked to explain the successes of SRS, SBRT/SABR, and IORT (23). Nevertheless, there is a strong case that large radiation doses may act as an adjuvant for immunogenic cell death by releasing tumor antigens and danger signals (24). At the same time, the identification and characterization of immune checkpoints has led to a surge in clinical studies on immune therapy using immune checkpoint blockade (ICB) antibodies (frequently termed “checkpoint inhibitors” although to date no small-molecule inhibitors are available). For example, an early phase II study showed dramatic effects in melanomas, which generally are immunogenic tumors (25). Thus, combining RT and ICB is considered to offer potential synergies, in particular since antitumor immune effects outside the irradiated target volume, so-called abscopal effects (26), might help control microscopic systemic disease.

Although the brain has, for decades, been regarded as a “privileged site” that provided limited scope for antitumor immunity, activated T cells are known to be able to cross the BBB (27). While conventional radiotherapy mildly increases BBB permeability (28), SRS disrupts the BBB within hours after application, allowing cells and substances to easily cross into the CNS for a period of roughly a month (29). In the case of BM, early studies suggested improved overall survival rates when ICB was combined with SRS for unresected metastases (30, 31), reaching levels similar to patients without BM (32). In contrast, a study applying ICB in patients previously treated with SRS found no significant difference to SRS alone (33) and ICB combined with conventionally fractionated WBRT after resection also failed to show an effect (34), suggesting that timing and fractionation may be important.

Whereas a potential interaction between SRS and ICB is readily understandable in the case of non-resected metastases where radiation can release tumor antigens, it is not obvious if the irradiation of residual tumor cells in the tumor bed after resection of the tumor is sufficient to elicit a tumor-directed immune response. Since no systematic studies on resected tumors have been published, a rationale for combining IORT with ICB must be based on an understanding of the mechanisms involved. Therefore, the purpose of the present review is to examine the immunological interaction between radiation and ICB to elucidate whether high single doses to the resection cavity and the residual cancer cells within its margins might act as an immunizing event opening the gate for ICB therapies in the brain. Because of the complexity and dynamic nature of this topic, we first give a brief overview of the antitumor immune response and immune checkpoints for the non-expert. We then present the experimental evidence for the interactions between radiation and the immune system. Finally, we review the clinical studies on SRS combined with ICB and discuss the implications and potential for combining IORT with ICB for BM.

## Activation of the Immune Response

The innate immune system acts as a non-specific first-line defense against infection and foreign antigens but also activates the adaptive immune system to provide an antigen-specific response. Upon infection, trauma (including irradiation), or during tumor initiation and progression (35), an inflammatory cascade is induced. In case of an infection, this is initiated by pathogen-associated molecular pattern (PAMP) molecules such as bacterial liposaccharides. Similarly, trauma release damage-associated molecular pattern (DAMP) molecules including proteins such as nuclear high-mobility group box 1 (HMGB1) and endoplasmatic calreticulin (CRT) as well as non-protein molecules adenosine triphosphate (ATP) and mitochondrial peptides and DNA (in the case of necrotic cell death) (36–38).

Soon after the appearance of PAMP or DAMP molecules, neutrophils enter the tissue secreting a large variety of chemokines and cytokines, including pro-inflammatory interleukin (IL)-12 (39), which in turn recruit monocytes and lymphocytes into the inflamed tissue. Depending on the cytokines, monocytes can differentiate into inflammatory or anti-inflammatory macrophages, and dendritic cells (DC). Phagocytes (neutrophils and macrophages) have pre-formed *pattern recognition receptors* (PRRs), mainly toll-like receptors (TLRs) and *receptors for advanced glycation end-products* (RAGE) that bind to PAMPs on microbial surfaces or to DAMPs from damaged cells. DAMPs are found on cell membranes, released into the extracellular space, or detected in the cytoplasm by intracellular PRR sensors such as TLR-9, which activates the STING [stimulator of interferon (IFN) genes] pathway (40) inducing the expression of type 1 IFN, e.g., IFNβ.

Natural killer (NK) cells are an important component of immune surveillance that remove cells with low expression of major histocompatibility complex (MHC) class I surface molecules. NK cells are CD3^−^ CD8^+^ lymphocytes lacking the T-cell receptor (TCR), which CD3^+^ lymphocytes use for the detection of antigens on MHC. Instead, they express activating receptors belonging to the family of killer-cell immunoglobulin-like receptors (KIRs). The body’s own cells are protected by inhibitory KIRs that recognize MHC class I presenting “self” antigens. Combinations of IL-12 or IL-15 with IL-18 stimulate NK cells activated by target cell recognition to secrete chemotactic cytokines, e.g., macrophage inflammatory protein followed by inflammatory cytokines IFNγ and tumor necrosis factor (TNF)-α in different subpopulations (41).

The adaptive immune system reacts to specific antigens and is made up largely of T and B lymphocytes, which are responsible for the cell-mediated and humoral adaptive immune responses, respectively. This part of the system carries a memory of previous antigens with lymphocytes being distributed between lymph nodes and the body tissues. Antigens need to be presented to lymphocytes by antigen-presenting cells (APCs). Most cell types present a small fraction of degraded proteins as peptide antigens on MHC molecules on their surface. Non-professional APCs (essentially all cell types) present 3–18 amino acid (a.a.) peptides from degraded cellular protein on 10^5^–10^6^ MHC I molecules found on each cell (42), while so-called professional APCs (DC found mainly in superficial tissue, macrophages, and B cells) also present peptides on MHC class II molecules. The peptides presented on MHC class II are generated from antigens taken up by endocytosis and can be longer than 18 a.a. but are often degraded by peptidases to approximately 12 a.a. (42). Tumor cells and dying normal cells translocate CRT to the cell surface acting as an “eat me” signal. If CRT is able to overcome the inhibitory “do not eat me” signal from CD47, it will activate TLRs on phagocytes (43, 44). Together with the release of other DAMP molecules, this stimulates phagocytosis by DC or macrophages which process the antigens and present them on MHC class II leading to activation of these APCs (45). Activated professional APCs migrate to the nearest lymph nodes (or *via* the blood vessels to the spleen) where the MHC:peptide complexes are presented to lymphocytes that recognize specific antigens by their T- or B-cell receptors (BCR). B cells recognize native antigens by their BCR and can internalize, process, and present antigen peptides on their MHC class II molecules to T cells (46). According to the clonal selection theory, the highly variable TCR and BCR give rise to an extremely large number of mature, so-called naive, lymphocytes that each recognize different epitopes made up of antigen peptides presented on MHC molecules. While an adaptive antitumor immune response requires CD8^+^ and CD4^+^ T cells, the role of B cells and the humoral adaptive immune response is unclear.

The two major classes of T cells, cytotoxic (“killer”) T cells (Tc) and helper T cells (Th), express different co-receptors, CD8 and CD4, respectively. CD8 on Tc bind to MHC class I (on all cells), while CD4 on Th cells bind to MHC class II (on professional APC). The binding between the Th and professional APCs is reinforced by induced expression of co-stimulatory molecules, mainly CD28, which binds to B7.1 (CD80) and B7.2 (CD86) on APCs, and CD40 ligand (CD40L), which binds to the CD40 receptor. Once a specific MHC II antigen-peptide combination binds the TCR and CD4 co-receptor of a naive Th, co-stimulatory binding results in its activation with clonal expansion and differentiation to a secretory effector Th cell releasing different cytokines.

**Figure 1:**
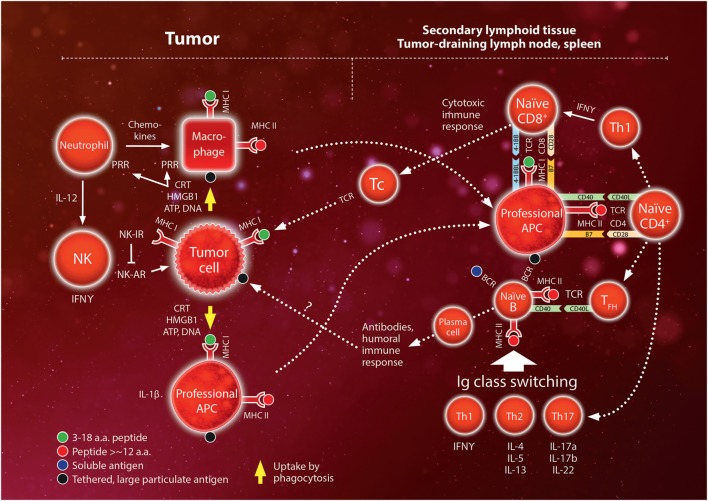
Schematic overview of the interaction between the innate and adaptive immune systems. The innate system initiates the immune response by reacting to pathogens or trauma. Pathogens release pathogen-associated molecular pattern molecules (e.g., liposaccharides) while trauma release damage-associated molecular pattern molecules [mainly calreticulin (CRT); high mobility group box (HMGB)-1; ATP; DNA]. These molecules bind to pattern recognition receptors (PRR) on phagocytes (neutrophils, macrophages). Neutrophils entering the tissue secrete a large variety of chemokines and cytokines which recruit monocytes and lymphocytes. Natural killer (NK) cells remove cells with low expression of major histocompatibility complex (MHC) class I surface molecules *via* a set of activating and inhibiting receptors (AR and IR, respectively). In the adaptive system, antigens are presented to lymphocytes by MHC molecules on antigen-presenting cells (APCs). All cells express MHC class I molecules but only professional APC (mainly dendritic cells, macrophages, and B lymphocytes) express MHC class II molecules. Professional APCs migrate to the secondary lymphoid tissue (lymph nodes and the spleen) where they activate naïve CD4^+^ and CD8^+^ T-lymphocytes. Depending on the cytokine expression of CD4^+^ T helper (Th) cells, these activated cells regulate class switching of naïve B lymphocytes to mediate the humoral immune response. Th1 also stimulate activation of CD8 cells to become cytotoxic (“killer”) T cells (Tc) that infiltrate the peripheral inflamed tissue and target specific antigens expressed on MHC class I molecules, e.g., on tumor cells. Interactions between MHC–antigen complexes and T cells are mediated by the T-cell receptor (TCR) and are reinforced by binding between pairs of complementary costimulatory molecules (e.g., B7 and CD28, CD40 and CD40 ligand, 4-1BB ligand and 4-1BB). Please also see text. For more detailed mechanisms, the reader is referred to comprehensive text books, e.g., Ref.(48, 49).

Subsets of differentiated Th cells mediate either a cytotoxic immune response (mainly Th1 cells characterized by secretion of IFNγ) or a humoral immune response (follicular helper, T_FH_) (47). Th1 cytokine IFNγ stimulates the function of macrophages and the activation of CD8^+^ T cells, binding to MHC I:peptide complexes. T_FH_ are thought to activate B cells, while Th1, Th2 (characterized by IL-4, IL-5, and IL-13), and Th17 (characterized by IL-17a, IL17b, and IL-22) direct immunoglobulin class switching according to different types of pathogens. Since B cells function as professional APCs they may activate Th cells recognizing the antigen peptides presented on the MHC II molecules of the B cell and the secreted cytokines in turn activate the B cell causing it to proliferate and produce specific antibodies. An overview of the immune activation is shown schematically in Figure 1 (48, 49).

## Immune Tolerance and Checkpoints

**Figure 2:**
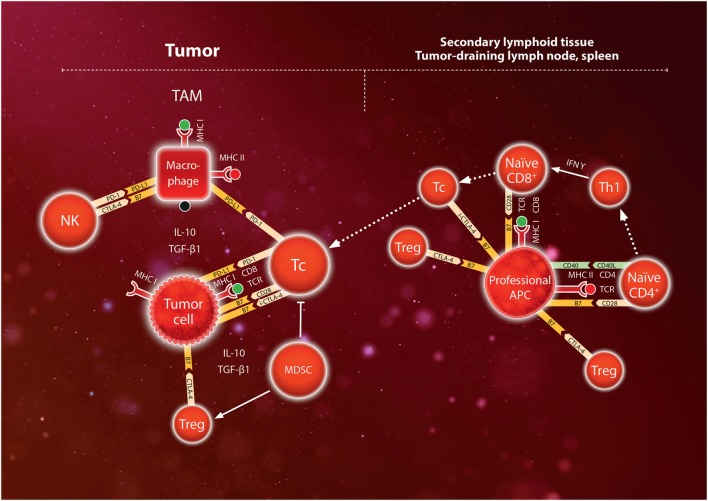
Schematic model of immunosuppressive mechanisms during T-cell activation in the secondary lymphoid tissue (lymph nodes or spleen) and during the anti-tumor immune response in the tumor. Naïve CD8^+^ lymphocytes express TCR which bind to a specific antigen presented by major histocompatibility complex (MHC) I on professional antigen-presenting cell (APC). Binding is reinforced by binding of CD8 to MHC, and secretion of IFNγ by Th1 cells leads to expression of the costimulatory molecules CD28 which binds to B7. Together, these signals activate the CD8^+^ lymphocyte to become a cytotoxic Tc lymphocyte. However, CTLA-4 on regulatory T cells (Treg) competes for B7 in the APC thus dampening T-cell activation. Furthermore, induced CTLA-4 (i-CTLA-4) may contribute to inhibiting the activity of Tc. Cytotoxic Tc lymphocytes infiltrate the tumor and engage tumor cells by binding of TCR to the MHC I antigen complex, which is reinforced by binding of costimulatory molecules CD28 to B7. However, myeloid-derived suppressor cells (MDSC) and tumor-associated macrophages (TAM) secrete IL-10 and TGF-β1 which stimulate Treg to express CTLA-4 competing for B7, and also directly inhibit Tc cells. Furthermore, tumor cells may upregulate expression of the programmed death (PD) ligand (L)1 which binds PD-1 on Tc thus inhibiting the activity of Tc against the tumor cells. In addition, TAM express PD-L1 binding to PD-1 on Tc and natural killer (NK) cells, and also B7 binding to CTLA-4 on NK cells. Tumor cells can upregulate these immune checkpoints to escape attack by the immune system. Use of immune checkpoint blockade (ICB) antibodies directed against CTLA-4 in the secondary lymphoid tissue or PD-1/PD-L1 in tumors can help override these immune checkpoints thereby stimulating immune activation (anti-CTLA-4) or inhibition of cytotoxic T-cells (anti-PD-1, anti-PD-L1).

Various mechanisms prevent the immune system from attacking its own body cells (autoimmune reactions) and from excessive normal immune reactions. Basically, tolerance to “self”-antigens is induced by the deletion of naive Tc recognizing MHC:peptide complexes that present fragments of the individuals own proteins. In addition, a number of other mechanisms help limit the physiological immune response. A special type of CD4^+^ regulatory T cells (Tregs, characterized by CD25^high^ and the canonical transcription factor FoxP3) limit or modulate the adaptive immune reaction by a variety of mechanisms [reviewed in Ref. (50)]. Tregs secrete inhibitory cytokines IL-10 and TGF-β1 and express CTLA-4 (cytotoxic T-lymphocyte-associated antigen 4) which is a negative regulator competing with CD28 for co-stimulatory binding to the B7 molecule on APCs [reviewed in Ref. (51)]. Tregs constitutively express CTLA-4 (52), but CTLA-4 is also induced during Tc activation, thus providing a feedback mechanism for downregulating APC-mediated Tc activation to prevent an excessive inflammatory reaction (53). Another member of the CD28 family, programmed death-1 (PD-1), is expressed on lymphocytes and inhibits the function of activated T cells, by binding to the B7 family ligand PD-L1. PD-L1 is not expressed in most normal cells but can be induced in tumor cells by IFNγ in the tumor microenvironment (54). PD-L1 can bind to B7.1, and PD-L1 signaling *via* PD-1 mediates immune suppression by stimulating apoptosis of T cells, inducing IL-10 and inducible Tregs, which contributes to a dysfunctional state termed T-cell “exhaustion” (55). Thus, according to the current model of immune checkpoints, CTLA-4 exerts its action mainly during antigen presentation and Tc activation, i.e., in the afferent arm of the adaptive immune response (leading to the secondary lymphoid tissue). By contrast, PD-L1/PD-1 is considered to act mainly in the efferent arm (leading from the lymph nodes back to the affected tissue) by modulating the cytotoxic action of CD8^+^ T cells in the tumor, although PD-1 is also expressed on Tregs, NK, and B cells, while PD-L1 is expressed on myeloid cells in tumors (56, 57). In addition to Tregs, myeloid-derived suppressor cells (MDSC; of monocytic and granulocytic lineages) contribute to immune suppression *via* secretion of immunosuppressive cytokines IL-10, and TGF-β1, and other mechanisms (58). The major immune checkpoints currently exploited in cancer therapy are shown schematically in Figure 2.

Because tumor cells arise from the body’s own cells they might be expected to escape immune surveillance. In spite of this inherent tolerance, an immune response may be elicited by overexpressing naturally occurring “self” proteins (tumor-associated antigens), mutated “self” proteins, or foreign proteins such as viral proteins (tumor-specific antigens, TSA) (59). However, genetic and epigenetic changes during tumor progression may select for mechanisms that avoid detection or suppress the immune response. Thus, an inflammatory response in tumors may upregulate PD-L1 and cause tumor-associated macrophages and MDSC to express IL-10 and TGF-β1 (60, 61).

Targeting the immune checkpoints by antibodies against CTLA-4 and PD-1/PD-L1 has recently shown to result in clinically relevant responses in some cancer patients (62–66). Antibodies against CTLA-4 broadly stimulate the adaptive immune response but may be associated with severe side effects, while anti PD-1/PD-L1 therapy may be more specific toward tumors and appears to be better tolerated (51). However, ICB antibodies given alone are effective only if the tumor is immunogenic *per se*.

## Radiation-Induced Enhancement of Immune Activity

Although low doses of radiation are immunosuppressive, it has become clear in the last 10–15 years that higher doses may stimulate the antitumor immune response (45, 67, 68). Indeed, some evidence suggests that immunogenic cell death contributes to the efficacy of hypofractionated or single-dose radiotherapy (37, 69, 70). However, data regarding the influence of dose and fractionation are conflicting, thus warranting a critical review of the dose dependence of immune activation.

The first evidence that irradiation releases DAMP molecules was found in murine thymoma cells that released HMGB1 after a dose of 10 Gy in an apoptosis-dependent fashion since the release was suppressed by the caspase inhibitor Z-VAD-fmk (71). Golden et al. found that CRT translocation and the release of DAMP molecules ATP and HMGB1 in a murine breast adenocarcinoma cell line were increased by single doses in the range of 2–20 Gy (72). The data indicated a quasi-linear increase up to 10 Gy, whereas 20 Gy produced a moderate further increase for CRT and ATP but only little further increase of HMGB1. Radiation-induced release of DNA into the cytosol (e.g., from the mitochondria) activates the STING pathway leading to the induction of type I IFN, the first step in the inflammatory cytokine cascade (73). Thus, IFNβ was induced after a single dose of 20 Gy to B16 melanoma tumors (74). NK cells and lymphocytes are very radiosensitive and undergo apoptosis after doses <2Gy. Furthermore, translocation of nuclear HMGB1 into the cytosol was recently reported after irradiation of human skin fibroblasts with doses in the range 4–12 Gy (75). Therefore, it seems a distinct possibility that high-dose irradiation of the normal tissue in the tumor bed may contribute to producing an inflammatory microenvironment conducive of an antitumor immune reaction.

Irradiation induces cytokines in various cell types, most importantly *via* nuclear factor (NF)-κB [reviewed in Ref. (76)], which can be activated by DNA damage-induced kinases, ATM, and DNA-PKcs (77, 78). Furthermore, HMGB1 is a ligand for RAGE and TLR4 signaling to NFκB, which may contribute to cytokine induction after higher doses (79). NFκB regulates transcription of a large number of cytokine genes, including pro-inflammatory cytokines such as IL-1β, IL-6, IL-8, IL-33, and TNF-α. Thus, expression of IL-1β and TNF-α was induced within a few hours of irradiating macrophages with doses of 3–20 Gy *in vitro* (80–82). Early upregulation of IL-1β was also observed after *in vivo* irradiation with 18.5 Gy (83), whereas a lower dose of 3 Gy caused upregulation approximately 5–7 days later, during macrophage differentiation preceding regeneration of the spleen (80). Early transcriptional upregulation of a number of cytokines including IL-1β and TNF-α occurred in brain or lung tissue after irradiation with doses of 7–25 Gy (84, 85). Thus, robust expression seems to require high single doses although daily fractions of 4 Gy also produced sustained expression in lung macrophages (85). Strong, dose-dependent secretion of IL-6 regulated by NFκB and activator-protein-1 was found in HeLa cells 24 h after irradiation with 3–20 Gy, while no significant increase was observed after 1 Gy (86, 87). In another study, secretion of IL1-α, IL-6, and IL-8 over 24 h was induced 1.7-, 1.6-, and 2.1-fold, respectively, by a low dose of 1.5 Gy in a monocytic cell line but not in A549 adenocarcinoma cells (88). However, irradiation of murine lymphoma with a single high-dose of 30 Gy initially decreased IFNγ and TNF-α in splenocytes but expression recovered 7–10 days after irradiation (70). A comprehensive review of the inflammatory response to ionizing radiation was given recently by Di Maggio et al. (89).

While it is not surprising that leukocytes express cytokines, it may be important for other cell types that p53 and NF-κB show a reciprocal relationship (90, 91). Veldwijk et al. (22) tested the secretion of 36 cytokines by p53 wild-type MCF7 breast cancer cells over 24 h after irradiation with 15 Gy. Only six cytokines (CD40L, IFNγ, IL-6, IL-8, IL-23, and Serpine E1) were detectable, and none showed significant upregulation after irradiation. Thus, it is conceivable that radiation-induced p53 may limit induction of the ATM/DNA-PKcs/NFκB pathway in p53 wild-type normal and tumor cells (A549 and MCF7) and that stronger induction in HeLa cells is due to the suppression of p53 by expression of the HPV18 E6 protein. Whereas *in vitro* induction may require high doses, there is ample evidence for radiation-induced expression of pro-inflammatory cytokines after moderate doses given *in vivo* (76). For example, dose-dependent upregulation was demonstrated in peritoneal mouse macrophages isolated 16 h after whole-body irradiation with 0.075–6 Gy with maximum upregulation at 4 Gy showing twofold increase for IL-12 and fivefold increase for IL-18 (92). The apparently higher sensitivity *in vivo* may be explained by additional activation due to lymphocyte apoptosis that may release DAMP molecules *in situ* including HMGB1 which activates the NF-κB pathway (79, 93).

Tumor cells frequently downregulate MHC surface molecules, thus reducing the opportunity of antigen presentation. However, radiation doses of 10–20 Gy upregulated MHC class I expression by ≥10% in 8/23 human colon, lung, and prostate, tumor cell lines tested (94). In a human melanoma cell line, MHC class I was increased in a dose- and time-dependent fashion with maximum expression at 48–72 h yielding a twofold increase for 10–25 Gy compared to 1.3-fold after 4 Gy (95). This study also showed that intracellular peptides for antigen presentation were initially generated by the degradation of existing proteins, but at later time points, novel peptides from new protein synthesis were presented on MHC class I. In a similar system, upregulation of MHC class I appeared to depend partly on radiation-induced IFNγ (96). Further aspects of different radiotherapy schemes on immune stimulation *in vitro* and *in vivo* have been reviewed in Ref. (97, 98).

Experiments using a tumor antigen-specific adenoviral vaccine showed that a single, moderate dose of 8 Gy given before vaccination produced a synergetic antitumor effect against a murine colorectal tumor, which was also observed when irradiation was given in three fractions of 3.5 Gy each (99). Since irradiation after vaccination had no effect, this seems to suggest a role of irradiation as an adjuvant creating a local microenvironment that supports immunization rather than a role in antigen presentation in this setting. Such a model is supported by the strong immunogenic effect of a TLR-7 agonist on a colorectal tumor, which was potentiated by fractionated radiotherapy with 5 × 2 Gy beginning simultaneously with the first application of the agonist but without any further immune therapy (100). However, the complexity and multiple components of the dynamic immune reaction may explain why combining radiotherapy with systemic type I or II IFNs was mostly unsuccessful, while the combination with IL-2 or IL-12 showed only limited effects in early clinical studies [reviewed in Ref. (101, 102)].

## Tumor-Directed, Radiation-Induced Immune Effects *In Vivo*

Few studies have investigated antitumor immunogenic effects of radiation *in vivo* without applying immune stimulation or checkpoint inhibitors. Lugade et al. found that a single radiation dose of 15 Gy increased the number of APC capable of activating IFNγ-secreting cells in lymph nodes in an experimental mouse model B16 of melanoma genetically modified to express ovalbumin (OVA) as a non-self antigen (67). A fractionated schedule of 5 × 3 Gy showed smaller increases of such APC in the lymph nodes. A similar difference between single and fractionated irradiation was seen for infiltration of the tumor by CD45^+^, CD4^+^, CD8^+^, CD11c, and CD11b immune cells 7 days after irradiation, indicating the recruitment of T cells, DC, macrophages, and possibly NK cells (CD8^+^ but CD3^−^). Interestingly, the difference between single and fractionated doses was observed for specific T cells, activated by tumor-derived peptide presentation on MHC I but not on MHC II in both lymph nodes and tumors. Infiltration into the tumors was due to lymphocyte trafficking and was dependent on the upregulation of vascular cell adhesion molecule-1 on endothelial cells (96). A study by Lee et al. using unmodified B16 melanoma confirmed a growth inhibitory effect after a single dose of 20 Gy when tumors were grown from 2 × 10^6^ injected cells, and local control was observed after 15 Gy when the number of injected cells was reduced to 1 × 10^5^ (69). In the same study, local tumor control was also observed when an MHC class I-binding peptide (“SIY”) was introduced as antigen and tumors grown from 2 × 10^5^ injected cells were irradiated with 25 Gy. Growth delay for 5 × 10^5^ injected cells and irradiation with 20 Gy was dependent on CD8^+^ and was not seen for fractionated irradiation with 4 × 5 Gy. The effect of dose and fraction size was studied by Schaue et al. who irradiated B16-OVA tumors with single doses of 5–15 and 15 Gy applied in 1, 2, 3, or 5, fractions (103). Inhibition of tumor growth was seen at 7.5–15 Gy, whereas no significant effect was seen after 5 Gy. Applying a dose of 15 Gy in 1, 2, 3, or 5, fractions reduced tumor size and increased antigen-specific IFNγ expressing cells in the spleen for all schedules with a trend for 2 × 7.5 Gy being more effective. Notably, doses of 1 × 7.5 Gy or 2 × 7.5 Gy, but not other doses, also seemed to reduce the number of Tregs in the spleen. Taken together, single doses of 15-25 Gy, or hypofractionated irradiation with large dose fractions (7.5 Gy), seem capable of eliciting an immunogenic antitumor response against the primary tumor in the B16 murine melanoma system even without including ICB in the treatment.

The combination of radiotherapy with ICB has shown considerable synergies on local tumor control in experimental systems. Demaria et al. found that a single dose of 12 Gy followed by CTLA-4 blockade showed a synergistic growth delay of mammary tumors and two fractions of 12 Gy separated by 48 h combined with CTLA-4 blockade produced local control in a small number of animals (104). In a study by Dewan et al., a single dose of 20 Gy, or daily fractionated irradiation with 3 × 8 Gy or 5 × 6 Gy, caused similar growth delay but adding anti-CTLA-4 antibody 2, 5, and 8 days after the first irradiation inhibited growth for all schemes with an apparent, small advantage of 3 × 8 Gy (105). Incidentally, 5 × 3 Gy fractionated irradiation of B16 melanoma produced slightly more tumor infiltration than a single dose of 15 Gy for CD8^+^ T cells activated by tumor-derived peptide presented on MHC class II, whereas 1 × 15 Gy produced higher numbers of cells activated by peptide presentation on MHC class I (67). This would seem consistent with a model in which hypofractionated irradiation combined with CTLA-4 blockade increases MHC class II-mediated antigen presentation by APC, while high single doses may be more efficient in promoting antigen presentation *via* MHC class I. In a radioresistant triple-negative mammary tumor studied by Verbrugge et al., a single dose of 12 Gy combined with anti-CD137 and anti-PD-L1 antibody treatment produced regression with some control of subcutaneous tumors while 4 × 5 Gy daily fractionated irradiation in combination with the same antibodies was effective in orthotopic tumors (106). Sharabi et al. showed regression of murine melanoma and mammary tumors irradiated with a single dose of 12 Gy combined with anti-PD-1 treatment (107). Irradiation with five fractions of 2 Gy upregulated expression of PD-L1 in colorectal cancer cells isolated from murine tumors but did not control tumors in a study by Dovedi et al. (108). However, concomitant administration of anti-PD-1 or anti-PD-L1 during and after irradiation resulted in 66–80% local control, and significant effects were confirmed in two other tumor lines. Irradiation combined with both anti-PD-1 and anti-PD-L1 showed no further enhancement. While local control was influenced by NK cells, survival was dependent on CD8^+^ T cells that also induced PD-L1 *via* IFNγ. Azad et al. irradiated syngenic pancreatic ductal adenocarcinoma (PDAC) tumors combined with anti-PD-L1 antibody therapy (109). In the KPC line, combined treatment produced non-significant growth delays after 1 × 6 Gy or 5 × 2 Gy, while a single dose of 20 Gy produced growth inhibition but excessive dermatitis required termination of the experiment. By contrast, combined treatment with a single dose of 12 Gy, or 5 × 3 Gy fractionated irradiation, caused significant growth delay in KPC and regression in the Pan02 line. This was associated with an increase in T-cell infiltration and a reduction in myeloid cell numbers and was only seen for simultaneous treatment and not when anti-PD-L1 was started 1 week after irradiation. In another study, Twyman-Saint Victor et al. showed that resistance in patients against hypofractionated SBRT combined with anti-CTLA4 was caused by the upregulation of PD-L1. Mimicking this in a mouse model, the resistance could be overcome by combining CTLA-4 and PD-L1 blockade with radiation, thus confirming and exploiting that the two immune checkpoints are non-redundant (110).

An overview of preclinical studies on immune effects in irradiated tumors is given in Table 1. Overall, dose fractions larger than 7–8 Gy seem to be more efficient in eliciting an inflammatory response and immune effects in irradiated tumors (67, 69, 103, 109). In many systems, tumor-infiltrating lymphocytes are increased after irradiation and an increase in the CD8^+^/Treg ratio seems to be associated with a successful immune reaction in some systems (103, 109, 110), although this is not universally found and MDSC reduction in tumors also seems to play a role (57, 110, 111). The question whether high single doses or hypofractionated irradiation with large fraction sizes is more efficient may depend on the tumor system, the role of antigen presentation by MHC class II, and the immune checkpoint being targeted.

**Table 1:** Preclinical results on the effect of immune reactions on the growth of the irradiated tumor.

Reference	Irrad. (RT)	Immunotherapy		Tumor model	Endpoint/effect/comments	
	no. fx, d/fx	Type	Start			
Lugade et al. (67)	1 × 15 Gy	None	n.a.	Melanoma (B16-OVA)	Activation of APC and specific immune cells, increased TIL trafficking	
	5 × 3 Gy	None	n.a.	Melanoma (B16-OVA)	Reduced growth delay, APC and MHC I-specific activation, TIL trafficking	

Lugade et al. (96)	1 × 15 Gy	None	n.a.	Melanoma (B16-OVA)	Radiation-induced IFNγ upregulates vascular cell adhesion molecule-1, MHC I	

Lee et al. (69)	1 × 20 Gy	None	n.a.	Melanoma (B16)	Growth delay	2 × 10^6^ cells inj.; delay T-cell dependent
	1 × 15 Gy	None	n.a.	Melanoma (B16)	Survival	1 × 10^5^ cells inj.; local control CD8^+^ dependent
	1 × 25 Gy	None	n.a.	Melanoma (B16-SYI)	Survival	2 × 10^5^ cells inj.
	1 × 20 Gy	None	n.a.	Melanoma (B16-SYI)	Growth delay	5 × 10^5^ cells inj., CD8^+^ dependent
	4 × 5 Gy	None	n.a.	Melanoma (B16-SYI)	No growth delay	5 × 10^5^ cells inj.

Schaue et al. (103)	1 × 15 Gy	None	n.a.	Melanoma (B16-OVA)	Growth delay	Signif. delay, activ. specif. splenocytes; (Treg) increased?
	1 × 10 Gy	None	n.a.	Melanoma (B16-OVA)	Growth delay	Signif. delay, activ. specif. splenocytes; (Treg reduced?)
	1 × 7.5 Gy	None	n.a.	Melanoma (B16-OVA)	Growth delay	Signif. delay, activ. specif. splenocytes; Treg reduced
	1 × 5 Gy	None	n.a.	Melanoma (B16-OVA)	Growth delay	No signif. delay, little splenocyte activ.; Treg unchanged
	1 × 15 Gy	None	n.a.	Melanoma (B16-OVA)	Growth delay	Signif. delay, activ. specif. splenocytes; (Treg increased?)
	2 × 7.5 Gy	None	n.a.	Melanoma (B16-OVA)	Growth delay	Signif. delay, activ. specif. splenocytes; Treg unchanged
	3 × 5 Gy	none	n.a.	Melanoma (B16-OVA)	Growth delay	Signif. delay, activ. specif. splenocytes; (Treg increased?)
	5 × 3 Gy	None	n.a.	Melanoma (B16-OVA)	Growth delay	Signif. delay, activ. specif. splenocytes; Treg increased (?)

Demaria et al. (26)	1 × 6 Gy	Flt3-L	1 day after	Breast ca. (67NR)	No enhanced growth delay (similar to RT)	
	1 × 2 Gy	Flt3-L	1 day after	Breast ca. (67NR)	No enhanced growth delay (similar to RT)	

Demaria et al. (104)	1 × 12 Gy	α-CTLA-4	1 day after	Breast ca. (4T1)	Growth delay	
	2 × 12 Gy	α-CTLA-4	1 day after	Breast ca. (4T1)	Regression/local control; tumor-specific CTL in spleen	

Dewan et al. (105)	1 × 20 Gy	α-CTLA-4	0 days	Breast ca. (TSA)	Growth delay	No regression
	1 × 20 Gy	α-CTLA-4	2 days after	Breast ca. (TSA)	Growth delay	No Regression
	3 × 8 Gy	α-CTLA-4	0 days	Breast ca. (TSA)	Growth delay	Regression
	3 × 8 Gy	α-CTLA-4	2 days after	Breast ca. (TSA)	Growth delay	Regression
	3 × 8 Gy	α-CTLA-4	4 days after	Breast ca. (TSA)	Growth delay	No Regression
	5 × 6 Gy	α-CTLA-4	2 days after	Breast ca. (TSA)	Growth delay	No regression
	1 × 20 Gy	α-CTLA-4	2 days after	Colon ca. (MCA38)	Non-signif. growth delay	
	3 × 8 Gy	α-CTLA-4	2 days after	Colon ca. (MCA38)	Growth delay	

Yoshimoto et al. (70)	1 × 30 Gy	None	n.a.	Lymphoma (EL4)	Survival	T-cell dependent
	1 × 30 Gy	None	n.a.	Lewis lung carc.	Growth delay	CD8^+^ dependent
	1 × 30 Gy	α-CTLA-4	1 day after	Lewis lung carc.	Growth delay	

Twyman-Saint Victor et al. (110)	1 × 20 Gy	α-CTLA-4	3 days before	Melanoma (B16-F10)	Growth delay	CD8^+^ dependent
	1 × 20 Gy	α-CTLA-4	1 day after	Melanoma (B16-F10)	Growth delay	
	1 × 20 Gy	α-CTLA-4, α-PD-L1	3 days before	Melanoma (B16-F10)	Survival	
	1 × 20 Gy	α-CTLA-4, α-PD-L1	3 days before	Breast ca. (TSA)	Survival	
	1 × 20 Gy	α-CTLA-4, α-PD-L1	3 days before	Pancreatic ca. (KPC)	Survival	
	1 × 20 Gy	α-CTLA-4, α-PD-1	3 days before	Melanoma (B16-F10)	Survival	

Verbrugge et al. (106)	1 × 12 Gy	α-CD40, α-CD137	0 days	Breast ca. (AT-3)	Growth delay	
	1 × 12 Gy	α-PD-L1	0 days	Breast ca. (AT-3)	Growth delay	
	1 × 12 Gy	α-CD137, α-PD-L1	0 days	Breast ca. (AT-3)	Growth delay	CD8^+^ depend., regression/control
	1 × 12 Gy	α-CD137, α-PD-L1	0 days	Orthopic AT-3	Survival	
	4 × 5 Gy	α-CD137, α-PD-L1	0 days	Breast ca. (AT-3)	Survival	
	4 × 4 Gy	α-CD137, α-PD-L1	0 days	Breast ca. (AT-3)	Regression	

Azad et al. (109)	1 × 20 Gy	α-PD-L1	0 days	Pancreatic ca. (KPC)	Growth delay	Termination due to dermatitis
	1 × 12 Gy	α-PD-L1	0 days	Pancreatic ca. (KPC)	Growth delay	CD8^+^ dependent
	1 × 12 Gy	α-PD-L1	6 days after	Pancreatic ca. (KPC)	No growth delay	
	1 × 6 Gy	α-PD-L1	0 days	Pancreatic ca. (KPC)	Growth delay	Non-significant, no regression
	5 × 3 Gy	α-PD-L1	0 days	Pancreatic ca. (KPC)	Growth delay	CD8^+^ dependent
	5 × 2 Gy	α-PD-L1	0 days	Pancreatic ca. (KPC)	Growth delay	Non-significant, no regression
	1 × 12 Gy	α-PD-L1	0 days	Pancreatic ca. (Pan02)	Regression	
	5 × 3 Gy	α-PD-L1	0 days	Pancreatic ca. (Pan02)	Regression	

Deng et al. (111)	1 × 20 Gy	α-PD-L1	1 day before	Colon ca. (MC38)	Regression	Delayed regrowth
	1 × 12 Gy	α-PD-L1	1 day before	Breast ca. (TUBO)	Regression	CD8^+^ dependent

Dovedi et al. (108)	5 × 2 Gy	α-PD-L1	1 day after	Colorectal ca. (CT26)	Survival	CD8^+^ dependent, CD4^+^ inhibits
	5 × 2 Gy	α-PD-1	1 day after	Colorectal ca. (CT26)	Survival	
	5 × 4 Gy	α-PD-L1	1 day after	Breast ca. (4T1)	Growth delay	
	5 × 2 Gy	α-PD-L1	1 day after	Myeloma (4434)	Growth delay	Delayed regrowth after regression

Sharabi et al. (107)	1 × 12 Gy	α-PD-1	1 day before	Melanoma (B16-OVA)	Regression	Treg in tumor increased by RT, but reduced by α-PD-1
	1 × 12 Gy	α-PD-1	1 day before	Breast ca. (4T1-HA)	Regression	Treg in tumor increased by RT, but reduced by α-PD-1

Park et al. (118)	1 × 15 Gy	α-PD-1	1 day before	Melanoma (B16-OVA)	Growth delay	
	1 × 15 Gy	α-PD-1	1 day before	Renal cell ca. (RENCA)	No enhanced growth delay (similar to RT)	

*α, anti; APC, antigen-presenting cells; TIL, tumor-infiltrating lymphocytes; MHC, major histocompatibility complex; Treg, regulatory T cells; VCAM-1, vascular cell adhesion molecule-1; PD-1, programmed death-1; PD-L1, PD-ligand 1*.

## Radiation-Induced Abscopal Effects

Sporadic cases of abscopal effects of radiotherapy were first described in clinical case reports (112–114), but meanwhile, this rare phenomenon is well documented although in some cases it may be associated (or to some extent overlap) with spontaneous regression [reviewed in Ref. (115)]. Experimentally, a non-specific abscopal effect on unirradiated distant tumors (Lewis lung carcinoma or T241 fibrosarcoma) was found by irradiating a non-tumor-bearing leg of mice with five fractions of 10 Gy each, whereas a lower dose of 12 × 2 Gy normo-fractionated irradiation was less effective (116). Interestingly, this effect was dependent on wild-type p53 function in the host animal cells. Irradiation and a special form of immunotherapy prevented distant metastases in the lung when primary tumors of a melanoma B16 line overexpressing CC chemokine receptor-7, or the breast cancer cell line 4T1, were irradiated with 2 × 12 Gy followed by adenoviral transduction with LIGHT, a TNF superfamily member, which enhances host immune responses (69). However, the systemic potential of radiation was much clearer when DC were stimulated by a growth factor or an ICB antibody was added (26, 105, 117). An early study achieved 60% long-term survival in a metastatic Lewis lung tumor model by irradiating the primary tumor with a single, very high dose of 60 Gy combined with the DC growth factor Flt-3 ligand (Ftl3-L) given for 10 days beginning 1 day after irradiation (117). Significant growth retardation was also obtained in a mammary tumor model after irradiation of one of the two tumors with a moderate dose of only 2 Gy combined with Flt3-L (26). In metastatic mammary tumors, the number of lung metastases was reduced in a CD8^+^-dependent fashion after 12 Gy followed by CTLA-4 blockade (104). Another study compared different fractionation schemes in combination with CTLA-4 blockade in irradiated primary and unirradiated secondary tumors (105). The growth delay in secondary tumors was larger for 3 × 8 Gy, intermediate for 5 × 6 Gy, and smallest for 1 × 20 Gy. For 3 × 8 Gy, delaying the CTLA-4 antibody until 4 days after the first fraction (2 days after the last fraction) reduced the abscopal effect. The alternative approach of combining radiation with a PD-L1 checkpoint inhibitor was tested using two mouse mammary tumors irradiated with single doses of 12 or 20 Gy combined with anti-PD-L1 every third day on days 0–9 (57). After regression of the primary tumor, rechallenge did not result in tumor growth, and furthermore, an abscopal effect on growth delay was seen in unirradiated secondary tumors. Similarly, blocking PD-1 at the time of irradiation showed abscopal effects on the growth of unirradiated secondary tumors (melanoma and renal cell carcinoma) when the primary tumors were irradiated with single fractions of 15 Gy (118). A recent study reported an anti-metastatic effect of radiation and anti-PD-L1 after *ex vivo* irradiation of tumor cells with 12 Gy but because no primary tumor was irradiated, this experimental design detected tumor take and not an abscopal effect (109). An overview of preclinical studies on abscopal effects of irradiation is given in Table 2.

**Table 2:** Preclinical results on abscopal immune effects (growth of non-irradiated secondary tumors) induced by irradiation elsewhere.

Reference	Irradiation	Immunotherapy		Irrad. tumor/abscopal	Abscopal endpoint/effect/comment	
			
	No. fx, d/fx	Type	Start	(Irrad. prim./unirrad. second.)	Non-irradiated tumor	
Camphausen et al. (116)	5 × 10 Gy	None	n.a.	Normal tissue/Lewis lung carc.	Growth delay	p53 dependent (host)

Lee et al. (69)	2 × 12 Gy	ad-LIGHT (transduct.)	0 days	Melanoma (B16-CC chemokine receptor-7)/n.a.	Metastases	1 × 10^5^ cells inj.
	2 × 12 Gy	ad-LIGHT (transduct.)	0 days	Breast ca. (4T1)/n.a.	Metastases	1 × 10^5^ cells inj.

Chakravarty et al. (117)	1 × 60 Gy	Flt3-L	1 day after	Lewis lung carc./metastases	Survival due to Tc dependent effect on metastases	

Demaria et al. (26)	1 × 6 Gy	Flt3-L	1 day after	Breast ca. (67NR/67NR)	Growth delay	
	1 × 2 Gy	Flt3-L	1 day after	Breast ca. (67NR/67NR)	Growth delay	T-cell dependent, tumor-specific

Demaria et al. (104)	1 × 12 Gy	α-CTLA-4	1 day after	Breast ca. (4T1/4T1)	Lung metastases reduced, CD8^+^ dependent	

Dewan et al. (105)	1 × 20 Gy	α-CTLA-4	0 days	Breast ca. (TSA/TSA)	No/insignif. growth delay			
	1 × 20 Gy	α-CTLA-4	2 days after	Breast ca. (TSA/TSA)	No/insignif. growth delay			
	3 × 8 Gy	α-CTLA-4	0 days	Breast ca. (TSA/TSA)	Reduced growth delay			
	3 × 8 Gy	α-CTLA-4	2 days after	Breast ca. (TSA/TSA)	Growth delay	
	3 × 8 Gy	α-CTLA-4	4 days after	Breast ca. (TSA/TSA)	More reduced growth delay			
	5 × 6 Gy	α-CTLA-4	2 days after	Breast ca. (TSA/TSA)	Intermediate growth delay			
	1 × 20 Gy	α-CTLA-4	2 days after	Colon ca. (MCA38/MCA38)	Non-signif. growth delay			
	3 × 8 Gy	α-CTLA-4	2 days after	Colon ca. (MCA38/MCA38)	Growth delay	

Yoshimoto et al. (70)	1 × 30 Gy	None	n.a.	Lymphoma (EL4/EL4)	No growth of second inoculation			
	1 × 30 Gy	None	n.a.	Lymphoma (EL4/EL4)	Growth delay of secondary tumor			

Twyman-Saint Victor et al. (110)	1 × 20 Gy	α-CTLA-4	3 days before	Melanoma (B16-F10/B16-F10)	Local control	

Deng et al. (111)	1 × 20 Gy	α-PD-L1	1 day before	Breast ca. (TUBO/TUBO)	Tumor rechallenge	
	1 × 12 Gy	α-PD-L1	1 day before	Breast ca. (TUBO/TUBO)	Growth delay of secondary tumor			

Park et al. (118)	1 × 15 Gy	None	1 day before	Melanoma (B16-OVA/B16-OVA)	Growth delay of secondary tumor; CD8^+^ dependent	
	1 × 15 Gy	α-PD-1	1 day before	Melanoma (B16-OVA/B16-OVA)	Growth delay of secondary tumor	
	1 × 15 Gy	α-PD-1	1 day before	Renal cell ca. (RENCA/RENCA)	Local control of secondary tumor, tumor specific	

α, anti; PD-1, programmed death-1; PD-L1, PD-ligand 1

Most studies found that immune effects of RT alone or in combination with ICB were dependent on CD8^+^ T cells (57, 69, 70, 94, 104, 106, 108). However, there is also evidence on an influence of NK cell (106, 108), though this has been less often tested and was not found in an earlier study (69). The role of CD4^+^ T cells is more ambiguous with little or even a negative influence in most studies (104, 106, 108), while an important role was reported in a glioma model (119). This variation may be explained by the fact that CD4^+^ represents not only tumor-reactive Th cells but also Treg cells. Since the latter constitutes a significant but variable fraction, the stimulating effect of Th and the inhibitory effect of Treg may frequently cancel each other. Although PD-L1 may enhance Treg, their number was not affected in the mammary tumors (57). Instead irradiation combined with anti-PD-L1 treatment was found to confer a delayed decrease in immunosuppressive MDSC mediated by TNF secreted by infiltrating Tc cells (57). Similarly, no change in the CD8^+^/Treg ratio but a late decrease in myeloid cell numbers was observed in PDAC tumors after irradiation with a single dose of 12 Gy combined with PD-L1 blockade (109).

In accordance with the stimulating effect of Flt3-L on antigen presentation and the effect of CTLA-4 inhibition on Tc activation and Treg downregulation, these agents were effective when applied concurrently with and immediately after irradiation though full abscopal effects were only manifested several weeks later. Since blocking the PD-1/PD-L1 checkpoint is considered to prevent the exhaustion of cytotoxic Tc lymphocytes infiltrating the tumor in the efferent phase, one might expect a synergistic effect by applying radiation and anti-PD-1/PD-L1 antibody sequentially. However, delaying the beginning of PD-L1 blockade until 6 days after irradiation abrogated the synergistic immune effect on irradiated tumors (109). Since four anti-PD-L1 treatments were given in 10 days, this seems to imply that irradiation acts on the tumor microenvironment *before* modulation by ICB, while ICB acts on the inflammatory microenvironment *induced* by irradiation. This suggests that although the PD-L1/PD-1 checkpoint is considered to be effective mainly in the efferent pathway of the adaptive immune response (120), it may be more important in the afferent pathway (activation and antigen presentation) after irradiation than previously thought. If this finding is confirmed in other systems, it would provide a strong argument for starting ICB immediately after irradiation (which is supported by initial clinical data, see below).

The success of ICB antibodies in preclinical and early clinical trials has prompted a large number of clinical trials applying different ICB antibodies with radiotherapy in different schedules and tumor sites [reviewed in Ref. (121)].

## Combining SRS with Immune Therapy for BM

With the discovery of a lymphatic vessel system in the CNS (122), and the knowledge that antigen presentation to T cells occurs in the (deep) cervical lymph nodes (123), it is becoming clear that the immune system of the brain communicates with its systemic counterpart (124). In fact the traditional concept of CNS immune privilege no longer seems appropriate (124, 125). Microglial cells representing CNS innate immune cells perform many functions similar to macrophages, including recognition of DAMP, while DC appear to be important for antigen presentation in the cervical lymph nodes (125). Thus, the general model of immune response and immunosuppression also applies to tumors located in the brain (126).

A series of articles by Lim and colleagues examined the interaction between stereotactic irradiation with a single dose of 10 Gy and different ICB antibodies in an intracranial glioma model using a small-animal irradiator. Anti-PD-1 antibody given three times in 4 days beginning the day of irradiation produced significant survival at 3 months in approximately 28% of the animals (127). Challenging the survivors with glioma cells in the flank demonstrated adaptive immune memory. Triple treatment with a CD137 agonist, an anti-CTLA-4 antibody, and radiation resulted in 50% long-term survival (119). Omitting the CD137 agonist yielded approximately 20% survival for concurrent treatment starting before or on the day of irradiation but only 10% when CTLA-4 inhibition was started 2 days after irradiation. Survivors after triple treatment also produced a memory response. A different triple treatment combining anti-TIM-3 and anti-PD-1 ICB antibodies with irradiation achieved 60% survival (128).

These preclinical data are in line with a number of clinical studies that suggested considerably improved overall survival rates by adding the antibody ipilimumab (IPI, anti-CTLA-4) to SRS (30–33, 129–131) (Table 3). In two of the studies, a median number of two BM was present (32, 131), but generally the number and size of metastases varied over a wide range. In some of the studies, information on prescription dose and fractionation was missing or incomplete but the treatment of individual BM with a single fraction of 20–21 Gy (median dose) appeared to be common (129, 131). However, doses and the number of fractions to individual BM varied: 14–24 Gy and 1–5 fractions (31), 15–20 Gy (129), 15–24 Gy in a single fraction (131), or 15–21 Gy with 16/20 patients receiving a single fraction and 3–5 fractions given to the last four (33). These early studies used retrospective or prospective series of patients, the sequence of IPI and SRS varied greatly, which may have contribute to the variable outcome, and frequently little detail was given regarding timing. Thus, clearly prospective studies with defined protocols are needed. Nevertheless, some of the studies seem to support the preclinical results that this ICB antibody shows better efficacy when given concurrently or immediately after SRS compared to delayed treatment although differences may exist between the irradiated metastases and abscopal effects on out-of-field disease (31, 33, 129, 131). However, although one trial included four patients who underwent prior resection of metastases before SRS to the cavity plus IPI (131), none have *a priori* addressed therapy of a purely resected population. Combining SRS with an anti-PD-1 antibody (nivolumab) has only been described in a single study on 73 lesions in 26 patients with median 9.4 months follow-up (132), including patients with resected lesions. Overall, local control (82% at 12 months) was comparable to conventional treatments, while distant control (53%) was higher than for other treatments. Interestingly, seven patients with resected BM appeared to have superior overall survival with five patients surviving after 24 months.

**Table 3:** Outcomes of combined application of stereotactic radiosurgery (SRS), and ipilimumab (IPI) in melanoma brain metastases (BM), whole-brain radiotherapy (WBRT).

Reference	Number of patients	Median OS	*P*
Knisely et al. (30)	50 (controls: SRS)	4.9 months	0.044
	27 (+IPI)	21.3 months	
	11 IPI before SRS	19.8 months	0.58
	16 IPI after SRS	21.3 months	

Silk et al. (31)	37 (controls: WBRT or SRS)	5.3 months	0.005
	33 (+IPI)	18.3 months	
	IPI before WBRT or SRS	8.1 months	n.a.
	IPI after WBRT or SRS	18.4 months	

Mathew et al. (129)	33 (controls: SRS)	45% 6-month OS	0.18
	25 (+IPI) before, concurrent, or after SRS	56% 6-month OS	

Shoukat et al. (130)	179 (controls: SRS)	6.8 months	<0.001
	38 (+IPI)	28.3 months	

Patel et al. (33)	34 (controls: SRS)	39% 1-year OS	0.84
	20 (+IPI)	37% 1-year OS	
	7 (+IPI) ≤ 15 days after SRS	43% 1-year OS	0.64
	13 (+IPI) > 15 days after SRS	34% 1-year OS	
	No IPI (SRS only)	39% 1-year OS	

Tazi et al. (32)	21 (no BM)	33.1 months	0.90
	IPI only (no SRS)		
	10 (BM, SRS)	29.3 months	
	+IPI concurrent or after SRS		

Kiess et al. (131)	IPI ≥ 9 weeks		
	15 IPI peri-/concurr. w. SRS (SRS during IPI)	65% 1-year OS	0.008
	12 IPI compl. before SRS (SRS > 1 month after IPI)	40% 1-year OS	
	19 IPI ≥ 1 day after SRS (SRS before IPI)	56% 1-year OS	

## Biological Effects of IORT

Although the application of radiotherapy during surgery to inactivate any malignant cells remaining after tumor excision is not a new concept, IORT has only become a practical option during the last decade owing to the development of novel, dedicated machines. Thus, mobile linear accelerators producing high-energy electrons, or miniature X-ray machines emitting LEX allow irradiation of the tumor bed in the operating room with minimal radiation protection issues directly after the tumor has been removed (133–135). Different dose distributions can be achieved using special applicators in combination with the type and energy of the beam (136–138). However, IORT differs from conventional adjuvant RT in several aspects that may potentially influence the biological effect [reviewed in Ref. (20, 21)].

Intraoperative radiotherapy is given as a single fraction during surgery, whereas fractionated RT has been the established procedure for decades, applying daily fractions of typically 1.8–2.0 Gy. Thus, IORT eliminates the time of some weeks required for wound healing between surgery and the beginning of RT during which residual cancer stem cells may proliferate and increase the number of recurrence-forming cells that need to be inactivated, or possibly spread by migration out of the tumor bed and thus escape focused SRS (139). SRS represents an intermediate between the two since it is usually applied as a single, large-dose fraction a few weeks after surgery. When comparing the biological effects of IORT and conventionally fractionated RT, the radiation quality, distribution of dose, and dose rate must be considered. High-energy electrons show a relative biologic effectiveness (RBE) similar to that of high-energy X-rays (20) and produce a relatively uniform dose distribution at dose rates of 1–5 Gy/min. IORT with LEX involves increased RBE values, a non-uniform dose distribution with a steep radial dose gradient, and protracted irradiation with reduced dose rates allowing the repair of sublethal damage during irradiation. The biological implications of these characteristics have been studied by radiobiological modeling and experimental measurements (140–142). Adverse reactions of the normal, healthy tissue are limited to a small volume around the applicator, while the risk of recurrence is predicted to be similar to that of conventional external beam radiotherapy within a spherical shell, the “sphere of equivalence,” thus defining a new target volume for tumor bed irradiation with LEX (140–145).

## Potential of Combining IORT with Immune Therapy for BM

The treatment of solitary BM by excision and IORT in 23 patients using 50 kV X-rays at a dose of 14 Gy in 2 mm depth yielded a disease-specific outcome at 5-year follow-up that was comparable to other modalities (18). In a large retrospective study from the same institution, localized RT versus WBRT alone or in combination was compared in 212 patients including 37 patients treated with SRS only and 19 patients treated with IORT only (146). The results indicated a slightly higher local recurrence rate for SRS/IORT, though this was not significant (*P* = 0.27). Rates of distant intracranial recurrences were higher than for local recurrences in both groups (WBRT and SRS/IORT) and were significantly higher after SRS/IORT compared with WBRT (*P* < 0.001). In spite of this, overall survival was comparable in the two groups and perhaps even marginally higher for SRS/IORT (*P* = 0.27). These results emphasize that distant recurrence is an issue when treating single lesions, especially with adjuvant localized RT although it may not directly affect overall survival.

At present, no studies combining IORT with ICB have been published. However, IORT differs from single fraction SRS by eliminating the delay between tumor excision and postoperative SRS. Thus, residual tumor cells are irradiated before they can be stimulated by factors released during the wound-healing process. Another important aspect is that the primary tumor is not irradiated but only the tumor bed, consisting mainly of normal brain tissue with an unknown, presumably low number of residual tumor cells. This poses the question whether the radiation-induced immune activity will suffice to elicit a tumor-directed immune response on which an interaction with ICB may be based. In the following, key points relevant to the potential use of ICB in combination with IORT for BM are discussed.

**Figure 3:**
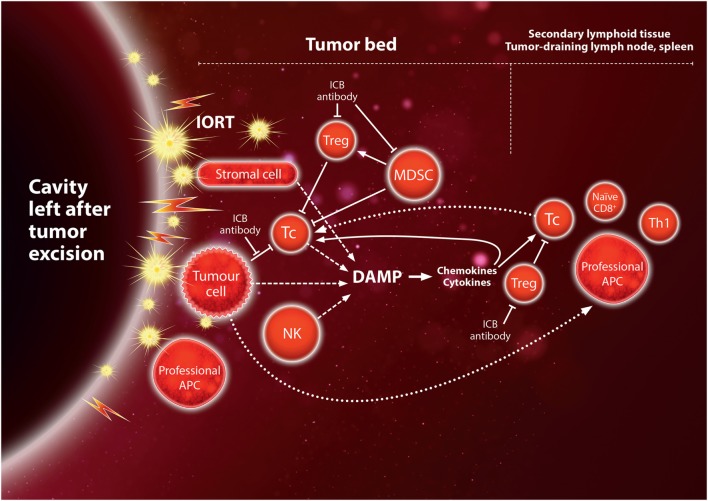
Hypothetical immune activation by IORT to the tumor bed after tumor excision of the metastasis. Irradiation of the normal tissue induces inflammatory “danger” signals, damage-associated molecular pattern (DAMP), leading to expression of chemokines and cytokines which recruit immune cells to the tumor bed (see also Figure 1), and may thus act as an adjuvant for the tumor-directed immune response. Cytotoxic Tc cells may target tumor cells as a result of being activated by antigen-presenting cell (APC) presenting tumor-specific or tumor-associated antigens before surgical excision. Immunogenic cell death of residual tumor cells in the tumor bed may contribute to antigen presentation and further inflammatory signals, creating a positive feedback loop. This would provide opportunities for synergy with immune checkpoint blockade (ICB) in the tumor bed or the secondary lymphoid tissue (see also Figure 2).

(1) *Safety and efficacy of IORT in BM*: a variety of reports have demonstrated that IORT is safe and efficient in primary [reviewed in Ref. (17)] and secondary brain tumors (18, 147, 148). The study from the Cleveland Clinic mentioned earlier yielded local control rates after surgery plus IORT similar to other modalities, despite including heavily pre-treated (SRS) recurrent BM (18). Of note, almost 60% of the patients died from extracranial progression. IORT as primary treatment after surgery would have the biological advantage of eliminating repopulation by remaining tumor cells during the delay between surgery and irradiation required for wound healing before SRS can be given. Based on the study on SRS plus nivolumab, in which neurotoxicity was mostly limited and could be relieved by treatment with steroids (132), combining IORT with anti-PD-1 is expected to be well tolerated.
(2) *Immunogenicity after resection of the tumor*: in contrast with most of the previous studies combining ICB with tumor irradiation, IORT is applied after the bulk of the tumor mass has been removed surgically. Therefore, only few tumor cells are expected to remain in the tumor bed after excision of the brain metastasis, raising the question whether the tumor load is sufficient to induce a tumor-directed immune response after irradiation. Since at least half of the patients will suffer local recurrence after surgery without adjuvant radiotherapy (1), tumor cells will be present in sufficient numbers to give rise to recurrence in these patients. Furthermore, antigens from the metastasis may already be presented to T cells by DC in the lymph nodes at the time of surgery. In addition, micrometastases elsewhere in the brain may contribute to an underlying endogenous immune response. The study on SRS plus nivolumab mentioned earlier showed an extended survival of 5/7 patients with resected metastases, whereas only 3/19 patients without resection were alive at 24 months (132). This strongly supports that irradiation of the resected cavity is indeed capable of eliciting an antitumor immune response and furthermore suggests that the tumor cell load may be an important factor in controlling residual disease. Further support that irradiation of normal tissue may play a role as an adjuvant in this response comes from the abscopal anti-tumor effect seen after irradiation of an unaffected leg with large fraction sizes (116). As discussed in the previous sections, preclinical studies indicate that the inflammatory microenvironment induced by high-dose irradiation may play an important role in enabling a tumor-directed immune response. Thus, while most irradiated lymphocytes in the tumor bed will undergo apoptosis after IORT, DAMP signals produced by irradiated immune cells and stromal cells, and cells damaged by the surgical procedure, may start a cascade of chemokines and cytokines that will attract and activate cells of the innate and adaptive immune systems. This will renew the lost lymphocyte population, which in turn may attack remaining tumor cells, thereby releasing more antigens and DAMP molecules.
(3) *Synergy between IORT and immunotherapy*: ICB antibodies in lymph nodes and the tumor bed – and to some extent irradiation itself – reduce the number and activity of immunosuppressive cells such as Treg and MDSC, thereby allowing a pre-existing antitumor immune response to become active. Thus, combining ICB antibodies with IORT is likely to enhance such a response. In this case, it is important to avoid irradiating (or exposing) the tumor-draining, deep cervical nodes, where antigen presentation to T cells may occur at the time of surgery since T cells are prone to undergo apoptosis even at moderate doses in the range 1–2 Gy. If breaking the immunosuppression is successful, an enhanced immune response to residual tumor cells may release more tumor antigens creating a positive feedback to reinforce the response (Figure 3). With development of a memory response, there may be a real chance for ICB in combination with IORT to establish a manifest abscopal immune response against microscopic disease elsewhere in the brain. Based on the majority of preclinical and clinical studies, it is likely that a single dose of at least 8 Gy high-energy photons (equivalent to approximately 6 Gy of LEX) will produce an immunogenic response and that ICB should be started simultaneously with irradiation. With 50 kV X-rays, such doses are feasible up to 8–10 mm from the surface of a spherical applicator. In the study on SRS plus nivolumab, the majority of patients received a single dose of 21–24 Gy SRS, although doses for patients with resected tumors were not specified. For IORT with 50 kV X-rays, doses in the range 14–20 Gy of 50 kV X-rays are achieved at 0–2 mm depth, corresponding to 18–27 Gy of high-energy X-rays when the higher RBE of 50 kV X-rays is taken into account [assuming RBE ~1.35 (142)].
(4) *Sequence of IORT and immunotherapy*: although to date, no systematic assessment on the sequence of application was performed, initial data point toward better outcome after concurrent application of SRS and immunotherapy. An analysis of 46 patients that received different schedules detected a trend toward better local control in patients receiving IPI during SRS (0% 1-year local recurrence) than in those receiving SRS before (13%) or after (11%) the administration of IPI (131). Similar data were shown in a retrospective analysis of 75 patients receiving SRS and anti-CTLA-4 or anti-PD-therapy: the study found that lesion responses were greater and more rapid with concurrent administration of immunotherapy and SRS (149). Translated into the setting of IORT, this would require administration of immunotherapy at the same day of surgery, provided that surgery is not complicated by the administration of the substances.
(5) *Safety of concurrent immunotherapy and surgery*: as concurrent application of immunotherapy and surgery appears to be required to achieve maximum therapeutic efficacy, safety is a major concern. Although not prospectively assessed, we believe that at least for the anti-CTLA4 antibody IPI and the anti-PD-1 antibody nivolumab, these concerns can be dispelled. Gyorki et al. analyzed 34 operations on 23 patients treated with IPI (150). Beside some grade 1 or 2 wound complications (22%), no grade 3–5 complications were seen. In line with this, a systematic review from Baker et al. also detected no IPI-related surgical complications so far (151). Similarly, neoadjuvant administration of nivolumab 2 or 4 weeks prior to surgery was seen to be safe and feasible in operable NSCLC (Forde et al. ESMO 2016, NCT02259621).

## Conclusion

Brain metastases have a high likelihood of local recurrence after resection, but at present, there is no standard radiotherapy technique to boost the surgical cavity. Thus, SRS to a narrow high-dose volume (e.g., by focusing different beam angles and/or by modulating the beam intensities) with Gammaknife or Cyberknife, or a linear accelerator are being used. An intraoperative boost of IORT appears a promising alternative, which does not require irradiating large volumes of healthy tissues or organs and which would eliminate the time required for wound healing (typically 2–4 weeks) before SRS is initiated. For both modalities, high single doses may elicit immunological effects that can reach beyond the tumor bed. A review of the mechanisms of radiation-induced immune reactions supports a model in which doses >~8 Gy may act as an adjuvant for antitumor immune reactions present before irradiation or enhanced by the release of tumor antigens from irradiated residual cancer cells in the tumor bed and possibly by immunogenic cancer cell death elsewhere. The efficacy of an immune response is supported by retrospective studies on SRS for (mainly) unresected BM combined with ICB antibodies (mostly IPI), suggesting that the antibody must be present at the time of and immediately after irradiation. Recent data on a small number of patients with resected BM indicate that SRS in combination with ICB antibodies, and in particular anti-PD-1, might increase overall survival in these patients, thus supporting the rationale for combining IORT with ICB for resected BM. Since IORT limits the dose to a small volume of normal brain tissue, one might even hypothesize that this approach would not preclude adding SRS in the case of oligometastases. Although these effects need to be more comprehensively understood, a combination therapy of very large dose fractions with ICB antibodies appears to be specifically synergistic, warranting further prospective clinical evaluation.

## Author Contributions

CH performed the literature search, wrote the manuscript, and drafted the figures. FW included clinical aspects and suggested literature. FG performed the clinical literature search and wrote the manuscript. All authors conceived of the aim of the review and read the final manuscript.

## Conflict of Interest Statement

Carl Zeiss Meditec AG, Jena, Germany, and Elekta, Crawley, UK, support training and radiobiological research at Universitaetsmedizin Mannheim. FG serves as a consultant and speaker for Carl Zeiss Meditec AG, NOXXON Pharma AG, Merck Serono GmbH, Roche Pharma AG, and Siemens Healthcare Diagnostics GmbH and holds patents related with Carl Zeiss Meditec AG. All other authors declare no conflict of interest.
